# Transcriptomic characterization of GMP-compliant regulatory macrophages (TRI-001) under inflammatory and hypoxic conditions: a comparative analysis across macrophage subtypes

**DOI:** 10.1186/s12967-025-06548-6

**Published:** 2025-05-16

**Authors:** Karina Zitta, Rouven Berndt, Katharina Hess, Fred Fändrich, Olga Gurvich, Katja Sirviö, Tuija Kekarainen, Lars Hummitzsch, Yuk Lung Wong, Ole Sattler, Steven Braem, Mark Krebs, Axel Fudickar, Sibille Engels, Nicole Flack, Markus Steinfath, Martin Albrecht

**Affiliations:** 1 Department of Anesthesiology and Intensive Care Medicine, University Hospital Schleswig– Holstein, Schwanenweg 21, Kiel, Germany; 2 Clinic of Vascular Medicine, University Heart and Vascular Center Hamburg, University Medical Center Hamburg-Eppendorf, Hamburg, Germany; 3 Vascular Research Center, University Hospital of Schleswig–Holstein, Kiel, Germany; 4 ICRSM Institute for Clinical Research and Systems Medicine, HMU Health and Medical University, Potsdam, Germany; 5 Department for Applied Cell Therapy, University Hospital Schleswig–Holstein, Kiel, Germany; 6 Ferring Ventures Oy, Kuopio, Finland; 7 3D-PharmXchange, Tilburg, The Netherlands; 8 Ferring Ventures GmbH, Hamburg, Germany; 9 Genevia Technologies Oy, Tampere, Finland

## Abstract

**Background:**

Regulatory macrophages (Mreg) represent a unique subset of macrophages known for their angiogenic and anti-inflammatory properties, positioning them as promising candidates for cell-based therapies. Recently, we have differentiated and characterized a distinct Mreg subtype (TRI-001), which is currently being produced in accordance with good manufacturing practice (GMP) for a multicenter study aimed at treating patients with peripheral arterial occlusive disease (PAOD).

**Aim of the Study:**

To compare the transcriptome of TRI-001 with various in vitro differentiated macrophage subtypes to provide a comprehensive context for TRI-001 within the macrophage landscape. Additionally, we aimed to develop a detailed transcriptome profile of TRI-001 under transient hypoxic and inflammatory conditions, mimicking the microenvironment in PAOD patients.

**Methods:**

Mreg were differentiated from human CD14 + monocytes using a GMP-compliant protocol and identified as TRI-001 by flow cytometry. Hypoxia was induced via an enzymatic model, while LPS treatment of TRI-001 was employed as inflammatory stimulus. Transcriptomic profiling was conducted using the Illumina HiSeq 4000 platform. In vitro cell migration assays (Oris assays) were conducted using human umbilical vein endothelial cells (HUVEC) cultured with supernatants derived from normoxia and hypoxia treated TRI-001.

**Results:**

TRI-001 demonstrated significant transcriptomic similarities with Mreg and Mreg_UKR but were different from M0, M1, M2a, and PCMO subtypes. Under hypoxic conditions and LPS stimulation, TRI-001 displayed distinct gene expression profiles compared to TRI-001 under control conditions, with hypoxic and LPS-stimulated profiles showing notable overlap. Pathway enrichment analysis suggested the activation of chemotaxis and migration-associated pathways especially under hypoxic conditions. Findings from functional in vitro cell migration assays were inconclusive, as the secretome of TRI-001, whether cultured under hypoxic or normoxic conditions, did not elicit a significant effect on endothelial cell migration.

**Conclusion:**

TRI-001 represents a novel type of regulatory macrophages (Mreg). The distinctive transcriptional responses to hypoxia and inflammatory stimuli highlight its potential as a cell therapy for the treatment of PAOD patients.

**Supplementary Information:**

The online version contains supplementary material available at 10.1186/s12967-025-06548-6.

## Introduction

Different macrophage subtypes such as regulatory macrophages (Mreg) can be in vitro differentiated from CD14 + monocytes using defined cell culture media [1]. The resulting cells have high clinical potential to be used as cell therapeutics, as they can positively impact angiogenesis and exhibit pro- or anti-inflammatory properties depending on the subtype [2, 3]. Accordingly, Mreg have already been successfully used in a clinical study to reduce rejection of kidney transplants [4, 5].

Over recent years, we have successfully differentiated and characterized a distinct type of Mreg with pro-angiogenic properties [6, 7]. These cells (TRI-001) are currently produced under GMP-compliant conditions and are planned for use in 2025 in a multicenter study for the treatment of patients suffering from peripheral arterial occlusive disease (PAOD; clinical trial number: Eudra CT 2024-517765-16). In the planned study, patients will receive 16 doses with a total of 40 million pro-angiogenic TRI-001 cells administered intramuscularly.

In contrast to our planned clinical trial, in studies of Riquelme and Hutchinson, Mreg cells (Mreg_UKR) were applied intravenously, and were detectable in various organs, such as the lungs, liver, spleen, and bone marrow, 30 h after administration [4, 5]. Although the authors could not describe any negative effects of Mreg_UKR in the respective organs, it remains largely unknown if and how organ-specific environments and microenvironments influence the differentiation and function of Mreg.

It is known that macrophages can alter their subtype and characteristics depending on the encountered microenvironment [6, 8, 9]. Since in the upcoming clinical trial TRI-001 cells will be administered intramuscularly in PAOD patients, they are expected to encounter a typical microenvironment, characterized by hypoxic and inflammatory regions [10]. The described intramuscular microenvironment could have a direct impact on TRI-001 by enhancing pro-angiogenic effects, such as the secretion of pro-angiogenic factors. On the other hand, the microenvironment could alter macrophage plasticity, potentially driving a phenotypic shift toward inflammatory M1 macrophages [8, 9].

In the current study we therefore compared the transcriptome of TRI-001 with that of other in vitro differentiated macrophage subtypes [1] to more precisely define the TRI-001 cell therapy product at the level of gene activity and to position it within the spectrum of different macrophage subtypes. Additionally, a detailed transcriptome profile of TRI-001 under transient hypoxic conditions and LPS treatment, reflecting the microenvironment typically found in PAOD patients, was generated to estimate the influence of these two conditions on TRI-001 gene expression and to gain initial insights into potential responses of TRI-001 after injection into human muscle tissue.

## Methods

### In vitro differentiation of TRI-001 and cell culture methods

**Fig. 1:**
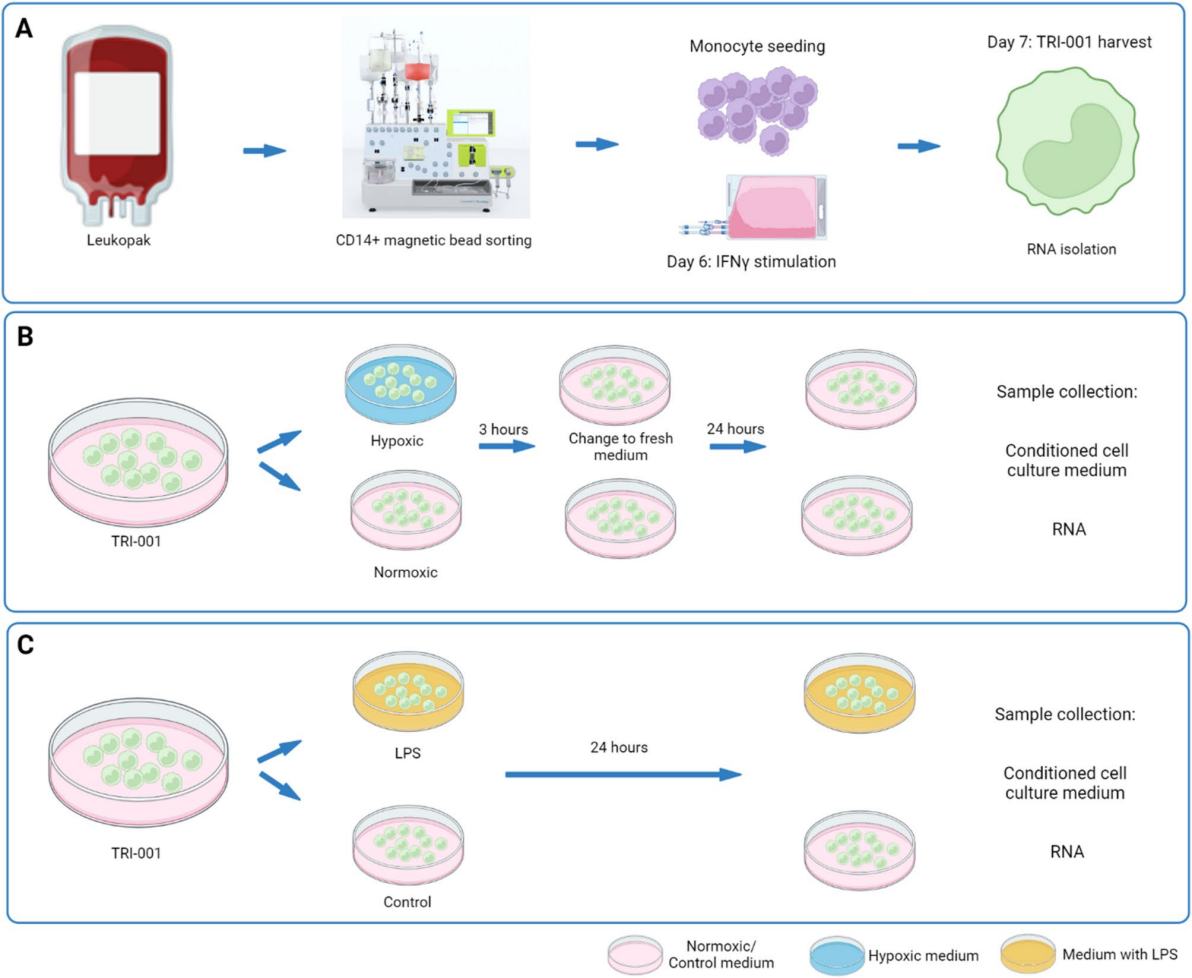
Schematic representation of the TRI-001 production process and outline of the cell culture experiments. CD14 + monocytes are isolated from leukopaks using magnetic bead sorting followed by differentiation into TRI-001 over a period of 7 days (**A**). Induction of hypoxia/reperfusion using an enzyme-based hypoxia model in vitro (**B**). Induction of inflammation using LPS stimulation in vitro (**C**)

The GMP-compliant production of Mreg (TRI-001), an advanced therapy medicinal product (ATMP), was conducted by Catalent (Gosselies, Belgium) using a modified version of our previously published protocol [6]. Key steps included enriching CD14 + monocytes from fresh leukopaks (Cellex, Cologne, Germany) via magnetic bead sorting using the Miltenyi Biotec CliniMACS Prodigy system (Miltenyi Biotec, Bergisch-Gladbach, Germany). Differentiation into TRI-001 was initiated by adjusting the rhM-CSF concentration to 2500 IU/mL and maintaining the cells at 34 °C until full differentiation was achieved (Fig. 1A). Three fully characterized batches of TRI-001 were used in all experiments conducted as part of the study (P34R020, P34R021 and 5550435).

In vitro hypoxia was induced following a two-enzyme system previously described by Hummitzsch et al. [11] to mimic ischemic conditions: The culture medium was replaced with medium containing 120 U/mL catalase (Sigma-Aldrich, Schnelldorf, Germany) and 2 U/mL glucose oxidase (Sigma-Aldrich) for 3 h, resulting in a reduced partial pressure of oxygen (pO₂; < 10 mmHg). In this model, also the pH and glucose levels gradually decrease, simulating conditions of the in vivo ischemic environment [12, 13]. For reoxygenation, hypoxic medium was replaced with fresh standard culture medium, resulting in an immediate restoration of physiological levels of pO₂, pH and glucose (Fig. 1B). Control experiments under normoxic conditions followed the same protocol omitting the hypoxia-inducing enzymes.

To induce an inflammatory stimulus, cells were incubated with 100 ng/mL lipopolysaccharide (LPS; Sigma-Aldrich) for 24 h (Fig. 1C). At the end of the differentiation period as well as after each treatment (hypoxia, normoxia, LPS, and control), conditioned culture media were collected, and RNA was isolated. For RNA extraction, 5-7.5 million cells were washed with PBS, lysed in 1 mL RLT buffer (Qiagen), and all samples were stored at -80 °C until further analysis.

### Flow cytometry

Flow cytometry analysis was conducted using the MACS Q10™ cytometer from Miltenyi Biotec. Specific antibodies and their corresponding isotypes, sourced from Miltenyi Biotec, were directly conjugated with phycoerythrin (PE) for CD66b; with PE-Vio615 for CD3 and CD38; with PE-Vio770 for CD19 and CD11c; with Viobright B515 for CD56 and CD31; with allophycocyanin (APC) for CD14 and CD206; with Vioblue for CD45 and near IR for live/dead cells. The gating strategy involved (i) identifying the CD45 population based on the size and granularity plot (FSC/SSC profiles), (ii) exclusion of doublets and non-viable cells and (iii) characterizing TRI-001 using the respective CD specific antibodies.

### RNA-sequencing samples and sequencing

Fifty-three samples were included in the analysis and are listed in Table S1. For the sixteen TRI-001 samples, generated in the current study, cells were lysed in 1 mL RLT buffer (Qiagen), and all samples were stored at -80 °C. RNA extraction and sequencing were performed by Eurofins Genomics (Luxembourg City, Luxembourg), generating approximately 30 million reverse-stranded 2 × 150 bp RNA-sequencing reads per sample on an Illumina HiSeq 4000 instrument. Data for the remaining 37 samples were generated previously by Gurvich et al. (2020) [1] using the same extraction method, library type, and service provider; raw reads for BioProject PRJNA552427 were downloaded in FASTQ format from the Sequence Read Archive (SRA) for inclusion in the analysis.

In the present study, TRI-001 cells were transcriptomically compared to several distinct macrophage subtypes, whose key characteristics are summarized as follows: Mreg cells represent anti-inflammatory macrophages generated by culturing monocytes with M-CSF and IFN-γ in flasks. Mreg_UKR cells are a variant produced in cultivation bags using a closely similar protocol. Although closely related at the transcriptomic level, they display distinct expression profiles due to differences in manufacturing. M0 macrophages are unstimulated and served as a neutral baseline. M1 cells, induced by IFN-γ and LPS, are pro-inflammatory and secrete high levels of IL-1β and TNF-α. M2a macrophages, stimulated with IL-4 or IL-13, are involved in tissue repair and express CD206 and IL-10. PCMO are monocyte-derived cells partially dedifferentiated under serum-rich conditions, exhibiting progenitor-like plasticity. For further information, please refer to [1].

### RNA-sequencing data analysis

For all samples, raw reads were trimmed to remove adapters and bases with a Phred score less than Q15 using *fastp* v0.23.1 [14]. Reads 15 bp and longer after trimming were retained for downstream analysis. Quality control results across samples were inspected with *MultiQC v2.3* [15].

Trimmed RNA-sequencing reads were aligned to the human reference genome assembly GRCh38 (GCF_000001405.26) and transcriptome annotation version GRCh38.99 using the *STAR* aligner v2.7.10a [16]. Gene-level read counts were obtained simultaneously during the alignment process using *STAR’s*–quantMode GeneCounts option. *MultiQC* v2.3 [15] was re-run to inspect the alignment results.

All downstream analyses were performed using R v4.2.2 (R Core Team, 2022). Data pre-processing, normalization, and exploratory clustering were performed using *DESeq2* v1.38.2 [18]; variance-stabilizing transformation (VST) was applied to normalized counts for exploratory visualization and clustering. Principal component analysis (PCA) was performed for the top 500 genes by sample variance across all samples and visualized with *ggplot2* v3.4.0 [19]. To evaluate broad transcriptional similarities among the sixteen cell types included in the analysis, hierarchically-clustered heatmaps were generated with *pheatmap* v1.0.12 [20] using Pearson’s correlation coefficients of the top 500 genes by variance as the distance metric and the default complete linkage agglomeration method.

For marker gene clustering the baseline TRI-001 transcriptome was characterized by comparing gene expression profiles to seven other cell types previously described by Gurvich et al. (2020) [1] (i.e., CD14 monocytes, M0, M1, M2a, Mreg, Mreg_UKR, and PCMO) via hierarchical clustering of 25 pre-selected marker genes. The complete list of marker genes is provided in Table S2. Hierarchical clustering of pre-selected marker genes was also performed with LPS-stimulated TRI-001, unstimulated control TRI-001, hypoxic TRI-001, and normoxic TRI-001 to identify changes in TRI-001 gene expression under inflammatory and ischemic conditions. Marker gene clustering and visualization were performed using the R package pheatmap v1.012 [20].

To further characterize changes in TRI-001 macrophage gene expression under hypoxic conditions, differential expression (DE) analyses were performed with DESeq2 version 1.38.2 [18]. Wald tests were used for assessing statistical significance of differential expression and p-values were adjusted for multiple testing using the Benjamini-Hochberg (BH) method [21]. Genes with BH-adjusted p-values < 0.05 and absolute log2 fold changes (L2FC) > 1 were deemed significantly differentially expressed.

Gene set enrichment analysis (GSEA) of Gene Ontology Biological Process (GO BP) terms was performed using TRI-001_Hypoxia vs.TRI-001_Normoxia log2 fold changes using R package clusterProfiler v4.9.0.2 [22]. Minimum and maximum gene set sizes were set to 15 and 500, respectively, and p-values were corrected for multiple testing using the Benjamini-Hochberg method [21]. Terms with an adjusted p-value < 0.1 were deemed significant. The top 20 pathways (either activated or suppressed based on normalized enrichment score) for TRI-001 Hypoxia vs. TRI-001 Normoxia were visualized in dotplots using the *enrichplot* R package v1.18.4 (Yu, 2023) [23].

### In-vitro cell migration assays (Oris assays)

Oris cell migration assays were conducted by Reaction Biology (Freiburg, Germany) using supernatants from three different batches of TRI-001 cultured under normoxic and hypoxic conditions. Primary human umbilical vein endothelial cells (HUVEC; PromoCell, #C-12203) were cultured and expanded in basal endothelial medium (PromoCell) supplemented with PromoCell supplement. HUVEC cells at passage 3 were used for the Oris assays.

Cells were harvested using Accutase (PAN Biotech), washed, and seeded at a density of 40,000 cells in 100 µL of complete medium (basal medium + supplements) per well in Collagen I-precoated Oris-96 well plates (AMS Bio). After 24 h, stopper inserts were removed, except for the zero controls (*n* = 3 per plate). The medium was then replaced with 100 µL of supernatants from TRI-001 cultured under normoxic and hypoxic conditions. Each plate included three wells as positive controls (complete medium) and three wells as negative controls (starvation medium, basal medium without supplements). After 24 h, the positive control wells with complete medium showed significantly greater cell coverage in the migration area compared to the controls.

Stoppers for the zero controls were removed, and the medium in all wells was exchanged for 75 µL of DMEM without phenol red (Gibco) containing 2 µg/mL Calcein-AM (Life Technologies). Cells were incubated for 15 min at 37 °C, and fluorescence in the insert-defined area was measured using a microplate reader (PerkinElmer, EnVision) with FITC settings (Ex: 485 nm/Em: 520 nm). Fluorescence data for each assay condition were recorded. The median fluorescence of the three zero controls (with stopper inserts) was subtracted from all assay values, and the data were then expressed as fluorescence in arbitrary units (a.u.).

### Statistics

In this work, a total of three different Mreg batches have been generated using a GMP compliant protocol. GraphPad Prism 10.1.0. for Windows (GraphPad Software, San Diego, USA) served as the statistical software for group comparisons. Prior to analysis, wound healing and cell migration data underwent Shapiro-Wilk testing for normality and were then subjected to one-way repeated measures ANOVA with Šídák correction for multiple comparisons. A p-value < 0.05 was considered statistically significant. Results are presented as mean ± standard error mean (SEM).

## Results

### TRI-001 display Mreg-specific cell surface markers

**Fig. 2:**
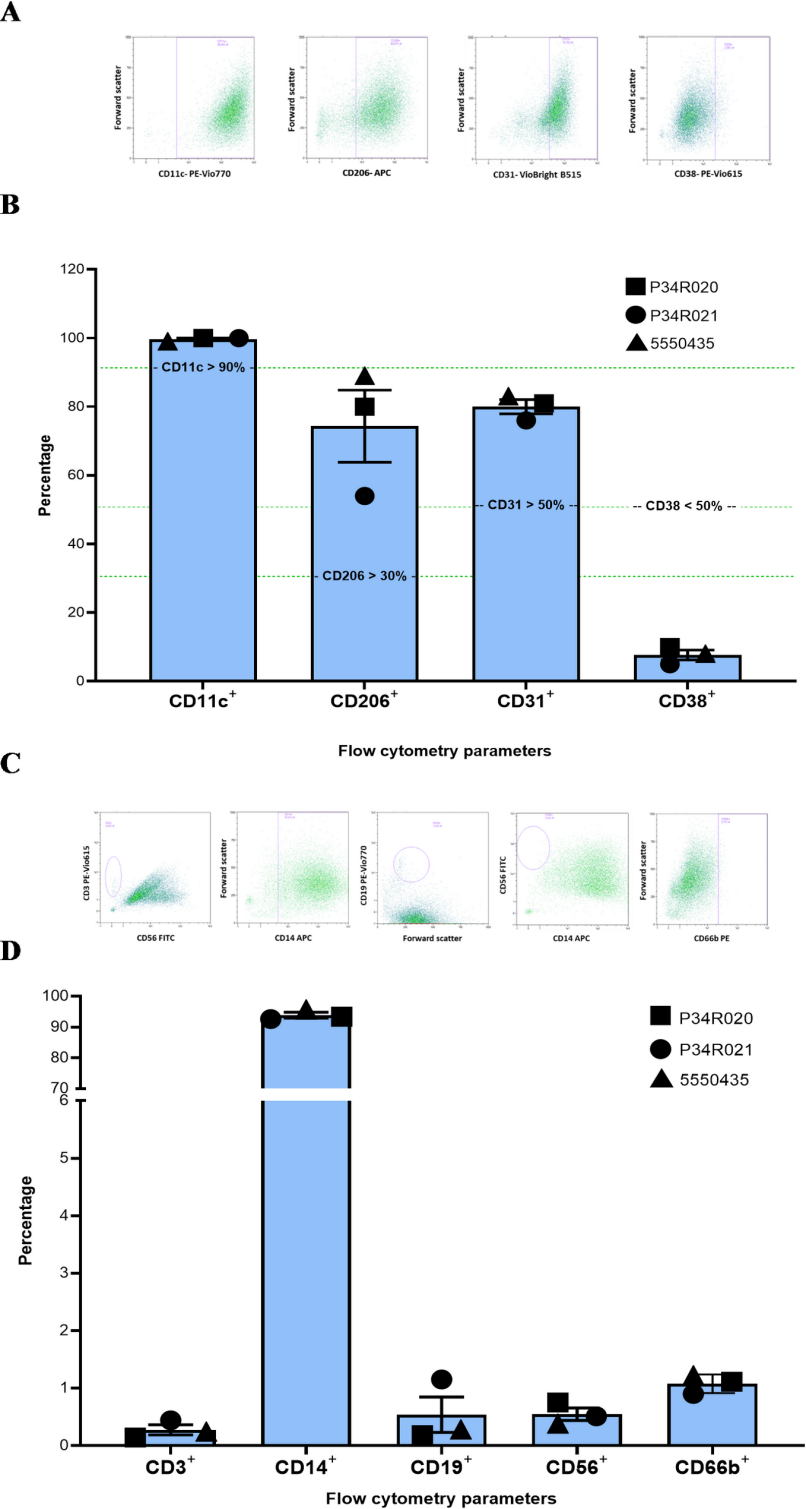
Flow cytometric quantification of identity and purity markers on TRI-001. The percentage of TRI-001 positive for the identity markers CD11c, CD31, CD206, and CD38 is shown for the three TRI-001 batches used in transcriptome analyses (**A** and **B**). The percentage of TRI-001 positive for the purity markers CD3, CD14, CD19, CD56 and CD66b is shown for the three TRI-001 batches used in transcriptome analyses (**C** and **D**). Bars represent the mean ± SEM. Horizontal lines indicate the defined thresholds for each marker

TRI-001 possess typical surface receptors (identity markers) that classify them as Mreg cells [1, 6]. All TRI-001 batches used in this study were subjected to flow cytometric analyses to ensure they met the defined identity marker expression relevant for Mreg and clinical application (CD11c > 90%, CD31 > 50%, CD206 > 30%, and CD38 < 50%). The percentage of cells positive for the corresponding marker was as follows: CD11c, 99.7 ± 0.6%; CD31, 80.0 ± 3.6%; CD206, 74.3 ± 18.2% and CD38, 7.7 ± 2.5%. Figure 2A and B provide an exemplary representation of the identity markers for the TRI-001 batches used for transcriptome analyses. In addition to identity markers, purity markers were analyzed to exclude cellular impurities such as T-lymphocytes (CD3+), B-lymphocytes (CD19+), Natural Killer Cells (CD56+) and Granulocytes (CD66b+) and to confirm the monocytic origin of TRI-001 (CD14+) (Fig. 2C and D).

### The transcriptome of TRI-001 aligns with Mreg while maintaining differences from other macrophage subtypes

**Fig. 3:**
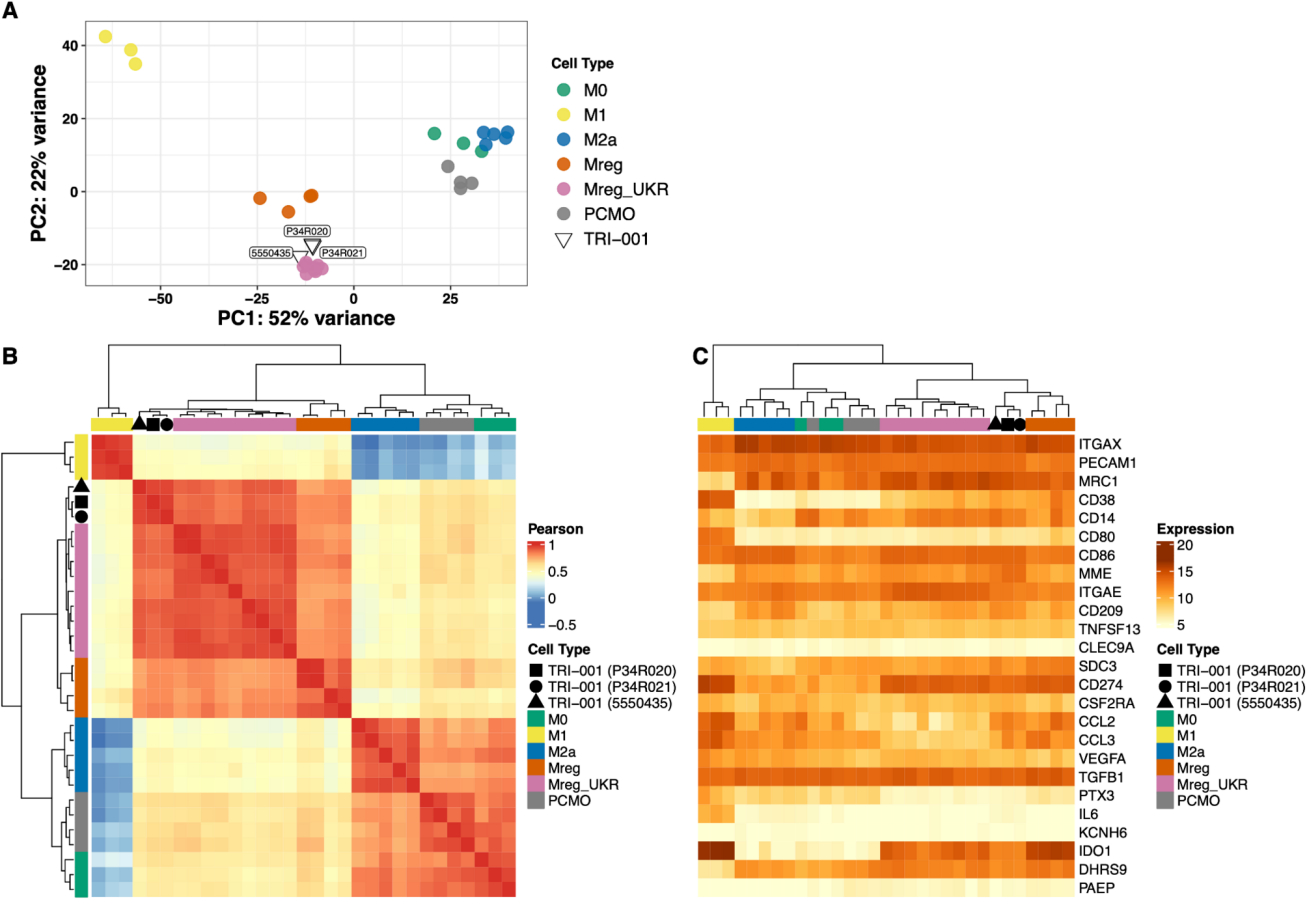
Comparison of baseline TRI-001 transcriptional landscape to selected macrophage subtypes [1]. Principal component analysis of the top 500 genes by sample variance of VST-transformed expression values. PC1 and PC2 are shown on the x- and y-axis, respectively. Groups are indicated with colors and the sources with symbols (**A**). Pairwise Pearson’s correlations on top 500 most variable genes. Samples are clustered using hierarchical clustering based on Pearson’s correlation coefficients. Groups and sources are annotated with colors/symbols on the top. Pearson correlations are visualized as a color scale from blue (low) to yellow (mid) to red (high) (**B**). Hierarchically clustered heatmap showing normalized expression of 25 pre-selected marker genes. Samples are clustered using hierarchical clustering based on VST-transformed expression values. Rows show genes and columns show samples. Normalized expressions are visualized as a color scale from yellow (low) to brown (high) (**C**)

For comprehensive, unbiased characterization of the cell product, TRI-001, the transcriptome was assessed by RNA-sequencing and compared to other in vitro differentiated macrophage subtypes [1] using principal component analysis (PCA). The first principal component explained 52% of the variance, primarily driven by differences between inflammatory M1 macrophages and anti-inflammatory, reparative M2a macrophages. The second principal component (PC2) accounted for 22% of the variance, with Mreg_UKR and M1 macrophages exhibiting the largest separation (Fig. 3a). Along PC1, TRI-001 were positioned midway between M1 macrophages on one side and PCMO, M0, and M2a macrophages on the other, suggesting functional distinctions of TRI-001 from these cell types. Instead, TRI-001 clustered with other regulatory macrophage types, including Mreg and Mreg_UKR. Minor variations within this cluster were observed, with TRI-001 and Mreg_UKR grouped closely together on one side, while Mreg formed a separate grouping (Fig. 3a).

The similarity between the samples was further investigated by creating a hierarchically clustered heatmap based on the Pearson correlation coefficients of each sample pair, calculated from the VST-transformed expression values of the top 500 genes by sample variance. The same clusters observed in the PCA analysis were maintained, with the first cluster containing only M1 macrophages, the second comprising Mreg, Mreg_UKR, and TRI-001, and the third including M0, M2a, and PCMO (Fig. 3b).

Additionally, the similarity of the samples was examined by generating a heatmap for 25 pre-selected marker genes, considering both key macrophage functions and Mreg-specific characteristics [6, 7, 11], to assess the similarity of the samples. The pre-selected marker genes are shown in Fig. 3c. While the identified separation of the macrophage subtypes into three distinct clusters was less pronounced here, the proximity of the various regulatory macrophage types (Mreg, Mreg_UKR, and TRI-001) remained evident (Fig. 3c).

### TRI-001 exhibit unique gene expression profiles under hypoxic and inflammatory conditions

TRI-001 will be injected into the ischemic muscle of PAOD patients as part of the planned cell therapy study. As a result, it is very likely that TRI-001 cells will be exposed to a hypoxic or inflammatory microenvironment. To assess how these conditions might influence the transcriptome of TRI-001, the relationship between the samples (TRI-001 Normoxia, TRI-001 Control, TRI-001 Hypoxia, and TRI-001 LPS) was investigated using principal component analysis (PCA).

**Fig. 4:**
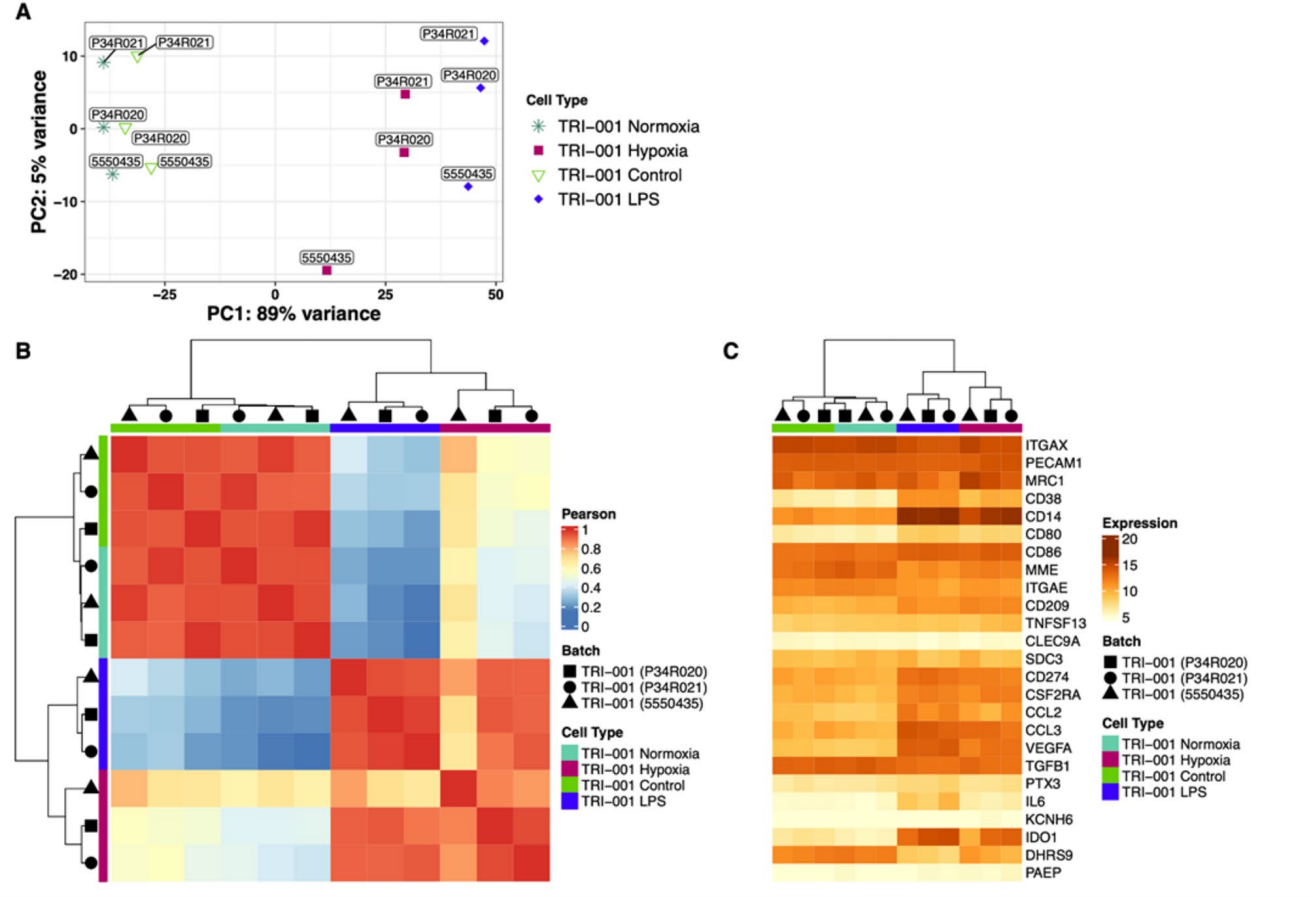
Comparison of TRI-001 gene expression under inflammatory and hypoxic conditions. Principal component analysis of the top 500 genes by sample variance of VST-transformed expression values. PC1 and PC2 are shown on the x- and y-axis, respectively. Groups are indicated with colors (**A**). Pairwise Pearson’s correlations of the top 500 most variable genes. Samples are clustered using hierarchical clustering based on Pearson’s correlation coefficients. Groups are annotated with symbols on the top. Pearson’s correlations are visualized as a color scale from blue (low) to yellow (mid) to red (high) (**B**). Hierarchically clustered heatmap showing normalized expression of 25 pre-selected marker genes. Samples are clustered using hierarchical clustering based on VST-transformed expression values. Rows show genes and columns show samples. Normalized expression values are visualized as a color scale from yellow (low) to brown (high) (**C**)

The first principal component (PC1 in Fig. 4a) explained 89% of the variance, primarily driven by the distinction between TRI-001 Normoxia and TRI-001 LPS. The second principal component (PC2) accounted for 5% of the variance, with TRI-001 Hypoxia and TRI-001 LPS showing the greatest separation. Focusing on PC1, two clusters were formed: one consisting of TRI-001 Normoxia and TRI-001 Control, and the other containing TRI-001 Hypoxia and TRI-001 LPS, suggesting that TRI-001 gene expression is notably altered under hypoxic and inflammatory conditions. However, there is also a substantial overlap in gene expression under hypoxia and inflammation (Fig. 4a).

The similarity of the samples was further explored by generating a hierarchically clustered heatmap of the Pearson correlation coefficients between each sample pair, based on expression of the top 500 most variable genes. The same clusters identified in the PCA were observed, demonstrating the highest similarity between TRI-001 Normoxia and TRI-001 Control, as well as between TRI-001 Hypoxia and TRI-001 LPS (Fig. 4b).

Additionally, the similarity of the samples was examined by generating a hierarchically clustered heatmap of the same 25 pre-selected marker genes as before (Fig. 3), considering both key macrophage functions and Mreg-specific characteristics [6, 7, 11]. The previously described separation of the analyzed macrophage subtypes into two distinct clusters was less pronounced here but still evident. Overlaps in the expressed genes between TRI-001 Hypoxia and TRI-001 LPS were observed. Notably, there was a significant increase in the expression of e.g. CD14, CD274 (PD-L1), CD38 and IDO1 in TRI-001 Hypoxia and TRI-001 LPS compared to the controls (Fig. 4c).

Further information on the samples analyzed in the study and the marker genes can be found in Table S1 and S2 of the Supplemental Materials. A Principal Component Analysis (PCA) of the top 500 most variable genes for all samples included in the analysis, as well as the pairwise Pearson’s correlations for the expression of the top 500 genes by variance for all samples (including CD14 + monocytes as starting material), are provided in Figure S3 and S4 of the Supplemental Materials.

### Pathway enrichment analysis suggests the activation of pathways linked to chemotaxis and migration in TRI-001 under hypoxic conditions

**Fig. 5:**
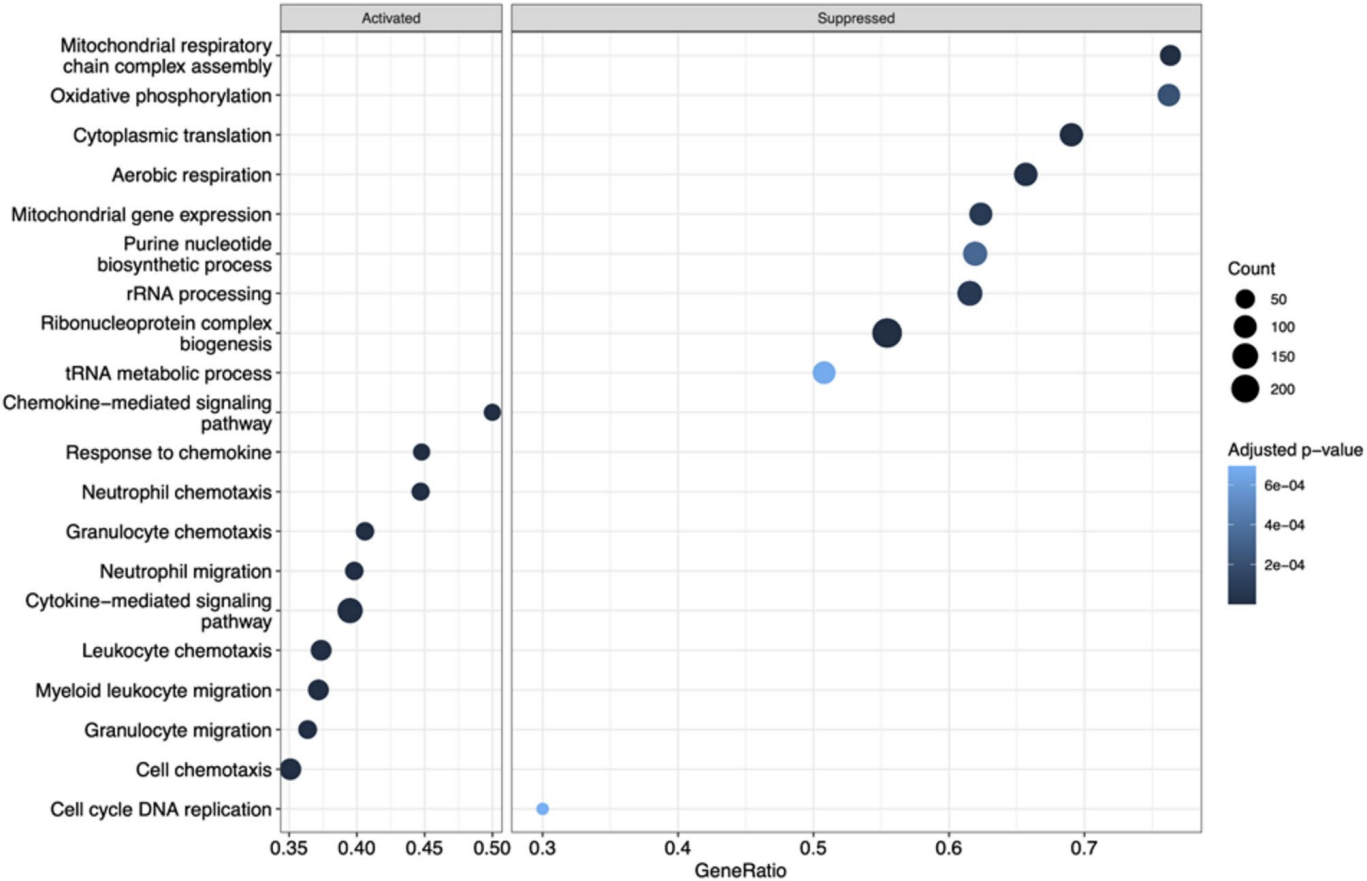
Dotplot representation of the top 20 most significantly enriched Gene Ontology Biological Pathways terms (both activated and suppressed) in comparison TRI-001 Hypoxia vs. TRI-001 Normoxia. X-axis shows the gene ratios of the enriched terms (i.e., the fraction of core enrichment genes vs. all genes associated with the pathway). Y-axis displays the enriched terms ordered by the gene ratio. Dot sizes depict the number of genes associated with the respective term. Dot color scale depicts the statistical significance of the enrichment, with dark blue and light blue indicating the lowest and highest adjusted p-values, respectively

To further characterize gene expression differences in TRI-001 under hypoxic vs. normoxic conditions, differential expression analysis was performed using DESeq2 and log2 fold changes were used as input for gene set enrichment analysis (GSEA). Pathway enrichment analysis was conducted via GSEA using the Gene Ontology Biological Process knowledgebase [24] and focusing on the top 20 activated and/or suppressed pathways by adjusted p-value. All 10 top pathways suppressed by hypoxia fell into the categories of protein synthesis (e.g., translation, ribosome biogenesis, rRNA processing) and cell metabolism (e.g., oxidative phosphorylation, aerobic respiration, mitochondrial respiratory chain complex assembly). In contrast, top pathways activated by hypoxia were associated with chemotaxis and cell migration (e.g., leukocyte chemotaxis, lymphocyte chemotaxis, granulocyte migration, neutrophil migration) suggesting that under hypoxic conditions, TRI-001 activate pathways involved in chemotaxis and cell migration - mechanisms that are also crucial for angiogenesis (Fig. 5).

### Secretory products from TRI-001 exposed to normoxia and hypoxia promote cell migration and support wound healing in vitro

**Fig. 6:**
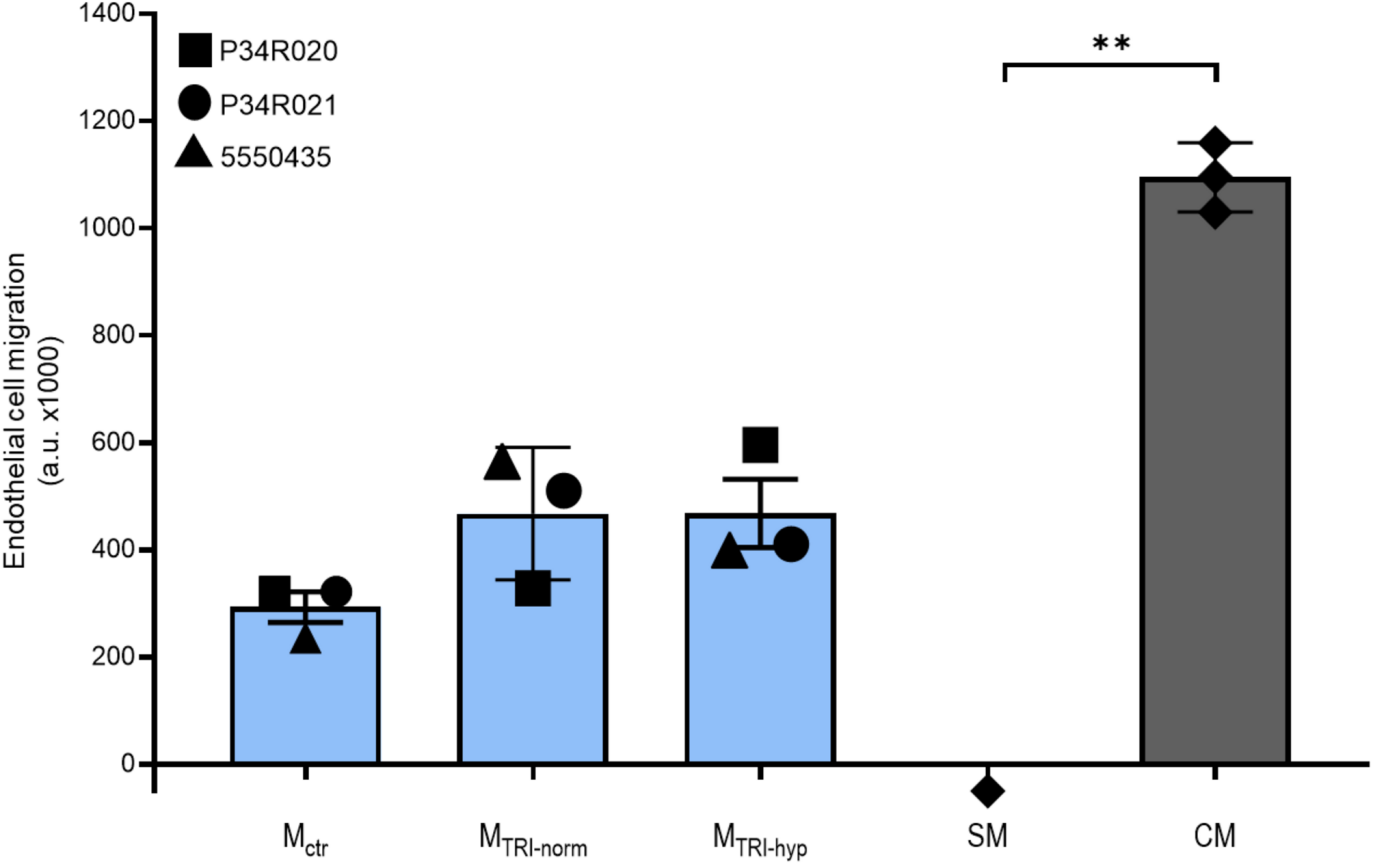
Effects of culture media from TRI-001 exposed to normoxic or hypoxic conditions on cell migration of endothelial cells. Analysis of the Oris assay using three different batches of TRI-001. Measurements were performed in triplicates for each batch. CM, complete medium (positive control); SM, starvation medium (negative control); M_ctr_, non-conditioned control medium; M_TRI_norm_, medium conditioned by normoxic TRI-001; M_TRI_hyp_, medium conditioned by hypoxic TRI-001; **, p < 0.01

Based on the pathway enrichment analysis data, which suggest that hypoxia may promote the activation of chemotaxis and cell migration pathways in TRI-001, Oris cell migration assays were conducted using endothelial cells to investigate a potential influence of the TRI-001 secretome on endothelial cell migration in vitro. The results demonstrated that, compared to non-conditioned control media (M_ctr_), media conditioned by TRI-001 under normoxic conditions (M_TRI_norm_) and media conditioned by TRI-001 under hypoxic conditions (M_TRI_hyp_) both had some positive effects on endothelial cell migration, although statistically significant levels were not reached: M_ctr_, 293.3 ± 28.67 a.u.; M_TRI_norm_, 467.5 ± 71.35 a.u.; M_TRI_hyp_, 468.0 ± 63.73 a.u. (M_ctr_ vs. M_TRI_norm_: *P* = 0.49; M_ctr_ vs. M_TRI_hyp_: *P* = 0.23 and M_TRI_norm_ vs. M_TRI_hyp_: *P* > 0.99; Fig. 6).

## Discussion

Macrophage subtypes differentiated from CD14 + monocytes hold great therapeutic potential, with pro- or anti-inflammatory as well as proangiogenic properties [1, 2, 4, 5, 11]. In the past years we have developed and characterized TRI-001, a pro-angiogenic Mreg subtype, manufactured in compliance with GMP, which will be employed in a multicenter trial to treat patients suffering from peripheral arterial occlusive disease (PAOD; clinical trial number: EudraCT 2024-517765-16).

In preparation for the clinical study, we aimed to compare the transcriptome of TRI-001 with various in vitro-differentiated macrophage subtypes to examine the macrophage profile of TRI-001 within the diverse spectrum of macrophage subtypes [1]. Additionally, we sought to establish a detailed transcriptome profile of TRI-001 under transient hypoxic conditions and LPS treatment as inflammatory trigger, simulating key aspects of the tissue microenvironment observed in PAOD patients.

Different macrophage subtypes exhibit distinct surface receptor expression, but their classification and function (e.g., M1-like or M2-like) are also influenced by intracellular signaling and secreted factors [1, 6, 8]. Based on our experience in characterizing regulatory macrophages [1, 6, 7, 11, 25], an identity marker panel was developed to define TRI-001. This, combined with various purity, release and potency markers/assays, ensures that consistency between TRI-001 batches is given for batches used in the clinical trial. For the present study, TRI-001 batches meeting all required criteria were selected, ensuring their representativeness for the corresponding cell therapy product.

Three typical batches of TRI-001 were then characterized by assessing their transcriptome by RNA-sequencing and comparing it to other in vitro differentiated macrophage subtypes described by Gurvich et al. [1].

Principal component analysis (PCA) revealed clear distinctions between the different macrophage subtypes. TRI-001 positioned between M1 and M2 macrophages, suggesting functional differences from both cell types, while clustering with other regulatory macrophages such as Mreg and Mreg_UKR. Hierarchical clustering and analysis of typical macrophage marker genes confirmed the separation of the macrophage subtypes into three distinct clusters, as seen in the PCA. The first cluster contained only M1 macrophages, the second cluster included Mreg, Mreg_UKR, and TRI-001, and the third cluster was composed of M0, M2a, and PCMO. The observed variations within the Mreg/TRI-001 cluster may be attributed to differences in the culture conditions used for differentiation process: TRI-001 and Mreg_UKR are differentiated in semipermeable cell culture bags, whereas Mreg cells are produced in culture flasks with two medium changes on days 1 and 4 during differentiation [1, 6, 7, 25]. Although the manufacturing processes for TRI-001 and Mreg_UKR are relatively similar, differences in the transcriptomes of these cell types are evident. These variations are likely due to differences in cultivation conditions, such as TRI-001 being cultured at 34 °C (compared to 37 °C for Mreg_UKR), and the amounts of M-CSF and IFN-gamma in the culture medium for TRI-001 production being adjusted based on their activity (U/ml), whereas for Mreg_UKR production, the concentrations of M-CSF and IFN-gamma in the culture medium are fixed (ng/ml) [1]. This finding supports previous reports that even small modifications in macrophage manufacturing protocols can impact the in vitro and possibly also in vivo function of the cells [1]. Consequently, for the production of macrophage-based cell therapy products, it is essential to apply GMP and rigorously characterize the final cell product, to achieve consistency of the product resulting in the desired effect of the cells in vivo and to minimize potential adverse reactions in patients.

In the context of our cell therapy study, TRI-001 will be injected into ischemic muscle tissue in PAOD patients, where they will possibly encounter hypoxic and inflammatory conditions [26–28]. To assess how distinct microenvironmental cues affect the TRI-001 transcriptome, we performed gene expression analyses under normoxia, hypoxia, LPS treatment (to induce a cellular inflammatory response), and standard control conditions. Principal component analysis (PCA) revealed two clusters: one containing TRI-001 Normoxia and Control and the other with TRI-001 Hypoxia and LPS. These findings were confirmed by hierarchical clustering and analysis of typical macrophage marker genes, suggesting significant gene expression shifts under hypoxic conditions and LPS stimulation as well as a degree of overlap in the response of TRI-001 when subjected to hypoxia or LPS.

A change in gene expression under hypoxic conditions and LPS stimulation is expected, and other authors have also shown that macrophages respond to hypoxia/ischemia and inflammation, resulting in significant changes in the transcriptome [29, 30]. However, in this context, the partial overlap in the transcriptome under hypoxia and LPS treatment is particularly interesting. For example, CD14, CD274 (PD-L1), CD38 and IDO1 showed increased expression under both hypoxic conditions and LPS stimulation compared to controls. These mentioned molecules are key factors involved in regulating immune responses, particularly in macrophages [31–34]. CD14 is a co-receptor for recognizing bacterial LPS, initiating immune responses [35], while CD274 (PD-L1) functions as an immune checkpoint, helping to suppress overactive T-cell responses [36]. CD38 is involved in NAD + metabolism, impacting energy balance in immune cells and supporting macrophage activation [37], whereas IDO1 mediates tryptophan metabolism, which leads to immune suppression by limiting T-cell proliferation [38]. Taken together these molecules play a crucial role in modulating macrophage functions, balancing immune activation with regulatory and tolerance mechanisms, and are important in contexts of both immune defense and suppression [37–39]. The observation that the expression of these factors is similarly regulated under both hypoxia and LPS stimulation suggests that hypoxia/ischemia and inflammatory stimuli, as seen in PAOD patients, may have partially overlapping effects on the applied cell therapy product, TRI-001.

Since TRI-001 will be injected into ischemic areas of the legs in PAOD patients, a key question is which signaling pathways are activated in TRI-001 under hypoxic conditions. Pathway enrichment analysis indicated that all hypoxia-activated pathways were related to chemotaxis and cell migration, suggesting that hypoxia activates TRI-001 pathways involved in chemotaxis and cell migration - mechanisms that are also essential for angiogenesis [40, 41].

Based on these results, Oris cell migration assays were performed to investigate the impact of the TRI-001 secretome on endothelial cell migration in vitro. The results of the assays indicate that media conditioned with TRI-001, under both normoxic and hypoxic conditions, tended to promote endothelial cell migration by over 30% compared to non-conditioned control media. Due to the small sample size, statistical significance was not achieved, and the results should be interpreted as a trend rather than definitive evidence. Nevertheless, these findings somewhat contrast with the results from the gene expression and pathway analyses, which show strong differences in gene expression between TRI-001 under normoxia and TRI-001 under hypoxia and suggest that pathways associated with cell migration and chemotaxis might play a role under hypoxic conditions. One possible explanation for this apparent discrepancy between gene expression analyses and cell migration/wound healing results is that numerous additional factors that were not analyzed within the scope of this study play a role in bridging the gap between gene expression, bioanalytical pathway assignment, and the actual in vivo function. Some examples are: Post-transcriptional modifications (e.g., mRNA splicing, microRNA regulation) [42], post-translational modifications (e.g., phosphorylation, glycosylation) [43–45], protein-protein interactions (e.g., inhibitors, activators), and protein stability and degradation (e.g., ubiquitination and proteasomal degradation) [46].

There are several limitations of this study that should be acknowledged: (i) Although the enzymatic hypoxia model used in this study closely mimics key aspects of tissue ischemia, it cannot fully recapitulate the complex and dynamic conditions encountered in vivo. As with all in vitro systems, it represents a compromise, and certain features of ischemia in patients - such as those seen in PAOD - may not be entirely reproduced. (ii) Lipopolysaccharide (LPS) was employed to induce an inflammatory response in the cell culture system. While this approach does not directly reflect the specific inflammatory milieu present in ischemic tissues of PAOD patients, it offers a controlled model to study general effects of a cell directed inflammatory trigger and its potential impact on TRI-001 behavior. This setup enabled the investigation of how an inflammatory stimulus might modulate cellular responses, although it is important to note that the specific inflammatory triggers in PAOD may differ, and the cells are unlikely to encounter LPS in vivo. (iii) The data presented in this study are largely based on transcriptomic analyses, which do not necessarily reflect the secretome of the cells. It should be noted, however, that substantial secretome data for TRI-001 and Mreg cells are already available [6, 11].

In summary, our data demonstrate that TRI-001 represents a novel and defined subtype of regulatory macrophages (Mreg) different from Mreg and Mreg_UKR. The unique transcriptional and functional responses of TRI-001 to inflammatory and hypoxic stimuli underscore their potential as a cell therapy for the treatment of PAOD patients.

## Electronic supplementary material

Below is the link to the electronic supplementary material.

Supplementary Material 1

## Data Availability

The datasets used and/or analysed during the current study are available from the corresponding author on reasonable request.
