# Supplementary material for: Transcriptomic characterization of GMP-compliant regulatory macrophages (TRI-001) under inflammatory and hypoxic conditions: a comparative analysis across macrophage subtypes
Source: J Transl Med. 2025 May 16;23:551. doi: 10.1186/s12967-025-06548-6 (PMC12085010; doi:10.1186/s12967-025-06548-6)
Supplement: Supplementary file 1 — Supplementary Material 1 [file 12967_2025_6548_MOESM1_ESM.docx]

## **Supplementary Material**

| \| File Name \| Sample Name \| Source \| Group \| \| --- \| --- \| --- \| --- \| \| NG-A1345_5550435_DP_con_libLAD9304_1.fastq.gz \| 5550435_DP_con \| Ferring Ventures \| TRI-001 LPS Control \| \| NG-A1345_5550435_DP_hyp_libLAD9303_1.fastq.gz \| 5550435_DP_hyp \| Ferring Ventures \| TRI-001 Hypoxia \| \| NG-A1345_5550435_DP_lps_libLAE5633_1.fastq.gz \| 5550435_DP_lps \| Ferring Ventures \| TRI-001 LPS \| \| NG-A1345_5550435_DP_nor_libLAD9302_1.fastq.gz \| 5550435_DP_nor \| Ferring Ventures \| TRI-001 Normoxia \| \| NG-A1345_5550435_DP_unt_libLAD9301_1.fastq.gz \| 5550435_DP_unt \| Ferring Ventures \| TRI-001 Untreated \| \| NG-A1345_5550435_MON_unt_libLAD9300_1.fastq.gz \| 5550435_MON_unt \| Ferring Ventures \| Monocyte Untreated \| \| SRR9851412_1.fastq.gz \| CD14_A \| Gurvich \| CD14 Untreated \| \| SRR9851416_1.fastq.gz \| CD14_B \| Gurvich \| CD14 Untreated \| \| SRR9851420_1.fastq.gz \| CD14_C \| Gurvich \| CD14 Untreated \| \| SRR9851389_1.fastq.gz \| CD14_D \| Gurvich \| CD14 Untreated \| \| SRR9851405_1.fastq.gz \| CD14_E \| Gurvich \| CD14 Untreated \| \| SRR9851400_1.fastq.gz \| CD14_F \| Gurvich \| CD14 Untreated \| \| SRR9851397_1.fastq.gz \| CD14_G \| Gurvich \| CD14 Untreated \| \| SRR9851392_1.fastq.gz \| CD14_H \| Gurvich \| CD14 Untreated \| \| SRR9851396_1.fastq.gz \| CD14_I \| Gurvich \| CD14 Untreated \| \| SRR9851415_1.fastq.gz \| M0_B \| Gurvich \| M0 Untreated \| \| SRR9851419_1.fastq.gz \| M0_C \| Gurvich \| M0 Untreated \| \| SRR9851425_1.fastq.gz \| M0_D \| Gurvich \| M0 Untreated \| \| SRR9851414_1.fastq.gz \| M1_B \| Gurvich \| M1 IFNg \| \| SRR9851422_1.fastq.gz \| M1_C \| Gurvich \| M1 IFNg \| \| SRR9851418_1.fastq.gz \| M1_D \| Gurvich \| M1 IFNg \| \| SRR9851406_1.fastq.gz \| M2a_E \| Gurvich \| M2a IL4 \| \| SRR9851401_1.fastq.gz \| M2a_F \| Gurvich \| M2a IL4 \| \| SRR9851398_1.fastq.gz \| M2a_G \| Gurvich \| M2a IL4 \| \| SRR9851391_1.fastq.gz \| M2a_H \| Gurvich \| M2a IL4 \| \| SRR9851395_1.fastq.gz \| M2a_I \| Gurvich \| M2a IL4 \| \| SRR9851411_1.fastq.gz \| Mreg_A \| Gurvich \| Mreg IFNg \| \| SRR9851413_1.fastq.gz \| Mreg_B \| Gurvich \| Mreg IFNg \| \| SRR9851421_1.fastq.gz \| Mreg_C \| Gurvich \| Mreg IFNg \| \| SRR9851417_1.fastq.gz \| Mreg_D \| Gurvich \| Mreg IFNg \| \| SRR9851410_1.fastq.gz \| Mreg_UKR_A \| Gurvich \| Mreg_UKR IFNg \| \| SRR9851408_1.fastq.gz \| Mreg_UKR_B \| Gurvich \| Mreg_UKR IFNg \| \| SRR9851424_1.fastq.gz \| Mreg_UKR_C \| Gurvich \| Mreg_UKR IFNg \| \| SRR9851403_1.fastq.gz \| Mreg_UKR_D \| Gurvich \| Mreg_UKR IFNg \| \| SRR9851399_1.fastq.gz \| Mreg_UKR_E \| Gurvich \| Mreg_UKR IFNg \| \| SRR9851402_1.fastq.gz \| Mreg_UKR_F \| Gurvich \| Mreg_UKR IFNg \| \| SRR9851393_1.fastq.gz \| Mreg_UKR_G \| Gurvich \| Mreg_UKR IFNg \| \| SRR9851390_1.fastq.gz \| Mreg_UKR_H \| Gurvich \| Mreg_UKR IFNg \| \| SRR9851394_1.fastq.gz \| Mreg_UKR_I \| Gurvich \| Mreg_UKR IFNg \| \| NG-A1345_P34R020_DP_con_libLAD9309_1.fastq.gz \| P34R020_DP_con \| Ferring Ventures \| TRI-001 LPS Control \| \| NG-A1345_P34R020_DP_hyp_libLAD9308_1.fastq.gz \| P34R020_DP_hyp \| Ferring Ventures \| TRI-001 Hypoxia \| \| NG-A1345_P34R020_DP_lps_libLAD9310_1.fastq.gz \| P34R020_DP_lps \| Ferring Ventures \| TRI-001 LPS Stimulated \| \| NG-A1345_P34R020_DP_nor_libLAD9307_1.fastq.gz \| P34R020_DP_nor \| Ferring Ventures \| TRI-001 Normoxia \| \| NG-A1345_P34R020_DP_unt_libLAD9306_1.fastq.gz \| P34R020_DP_unt \| Ferring Ventures \| TRI-001 Untreated \| \| NG-A1345_P34R021_DP_con_libLAD9314_1.fastq.gz \| P34R021_DP_con \| Ferring Ventures \| TRI-001 LPS Control \| \| NG-A1345_P34R021_DP_hyp_libLAD9313_1.fastq.gz \| P34R021_DP_hyp \| Ferring Ventures \| TRI-001 Hypoxia \| \| NG-A1345_P34R021_DP_lps_libLAD9315_1.fastq.gz \| P34R021_DP_lps \| Ferring Ventures \| TRI-001 LPS Stimulated \| \| NG-A1345_P34R021_DP_nor_libLAD9312_1.fastq.gz \| P34R021_DP_nor \| Ferring Ventures \| TRI-001 Normoxia \| \| NG-A1345_P34R021_DP_unt_libLAD9311_1.fastq.gz \| P34R021_DP_unt \| Ferring Ventures \| TRI-001 Untreated \| \| SRR9851409_1.fastq.gz \| PCMO_A \| Gurvich \| PCMO IL3 \| \| SRR9851407_1.fastq.gz \| PCMO_B \| Gurvich \| PCMO IL3 \| \| SRR9851423_1.fastq.gz \| PCMO_C \| Gurvich \| PCMO IL3 \| \| SRR9851404_1.fastq.gz \| PMCO_D \| Gurvich \| PCMO IL3 \|   *Table S1: Samples analysed in the current study. Raw sequencing reads for samples generated by Gurvich et al. (2020) were downloaded from the NCBI Sequence Read Archive (SRA), BioProject PRJNA552427. All samples were sequenced by Eurofins Genomics (Luxembourg City, Luxembourg) to generate approximately 30 million reverse-stranded 2 x 150 bp RNA-sequencing reads per sample on an Illumina HiSeq 4000 instrument.* |
| --- | --- | --- | --- | --- | --- | --- | --- | --- | --- | --- | --- | --- | --- | --- | --- | --- | --- | --- | --- | --- | --- | --- | --- | --- | --- | --- | --- | --- | --- | --- | --- | --- | --- | --- | --- | --- | --- | --- | --- | --- | --- | --- | --- | --- | --- | --- | --- | --- | --- | --- | --- | --- | --- | --- | --- | --- | --- | --- | --- | --- | --- | --- | --- | --- | --- | --- | --- | --- | --- | --- | --- | --- | --- | --- | --- | --- | --- | --- | --- | --- | --- | --- | --- | --- | --- | --- | --- | --- | --- | --- | --- | --- | --- | --- | --- | --- | --- | --- | --- | --- | --- | --- | --- | --- | --- | --- | --- | --- | --- | --- | --- | --- | --- | --- | --- | --- | --- | --- | --- | --- | --- | --- | --- | --- | --- | --- | --- | --- | --- | --- | --- | --- | --- | --- | --- | --- | --- | --- | --- | --- | --- | --- | --- | --- | --- | --- | --- | --- | --- | --- | --- | --- | --- | --- | --- | --- | --- | --- | --- | --- | --- | --- | --- | --- | --- | --- | --- | --- | --- | --- | --- | --- | --- | --- | --- | --- | --- | --- | --- | --- | --- | --- | --- | --- | --- | --- | --- | --- | --- | --- | --- | --- | --- | --- | --- | --- | --- | --- | --- | --- | --- | --- | --- | --- | --- | --- | --- | --- | --- | --- | --- | --- | --- | --- | --- | --- |

| \| Gene Symbol \| Gene Aliases \| Ensembl ID \| \| --- \| --- \| --- \| \| CSF2RA \| GM-CSFR; CD116 \| ENSG00000198223 \| \| CSF2RB \| CD131 \| ENSG00000100368 \| \| CCL2 \| - \| ENSG00000108691 \| \| CCL3 \| - \| ENSG00000277632 \| \| VEGFA \| - \| ENSG00000112715 \| \| TGFB1 \| - \| ENSG00000105329 \| \| PTX3 \| - \| ENSG00000163661 \| \| IL6 \| - \| ENSG00000136244 \| \| ITGAX \| CD11C \| ENSG00000140678 \| \| PECAM1 \| CD31 \| ENSG00000261371 \| \| MRC1 \| CD206 \| ENSG00000260314 \| \| CD38 \| - \| ENSG00000004468 \| \| CD14 \| - \| ENSG00000170458 \| \| CD80 \| - \| ENSG00000121594 \| \| CD86 \| - \| ENSG00000114013 \| \| MME \| CD10 \| ENSG00000196549 \| \| ITGAE \| CD103 \| ENSG00000083457 \| \| CD209 \| - \| ENSG00000090659 \| \| TNFSF13 \| CD258 \| ENSG00000161955 \| \| CLEC9A \| CD370 \| ENSG00000197992 \| \| SDC3 \| - \| ENSG00000162512 \| \| CD274 \| PD-L1 \| ENSG00000120217 \| \| KCNH6 \| ERG2 \| ENSG00000173826 \| \| IDO1 \| IDO \| ENSG00000131203 \| \| DHRS9 \| - \| ENSG00000073737 \| \| PAEP \| - \| ENSG00000122133 \|   *Table S2: Marker genes by category.*   \| *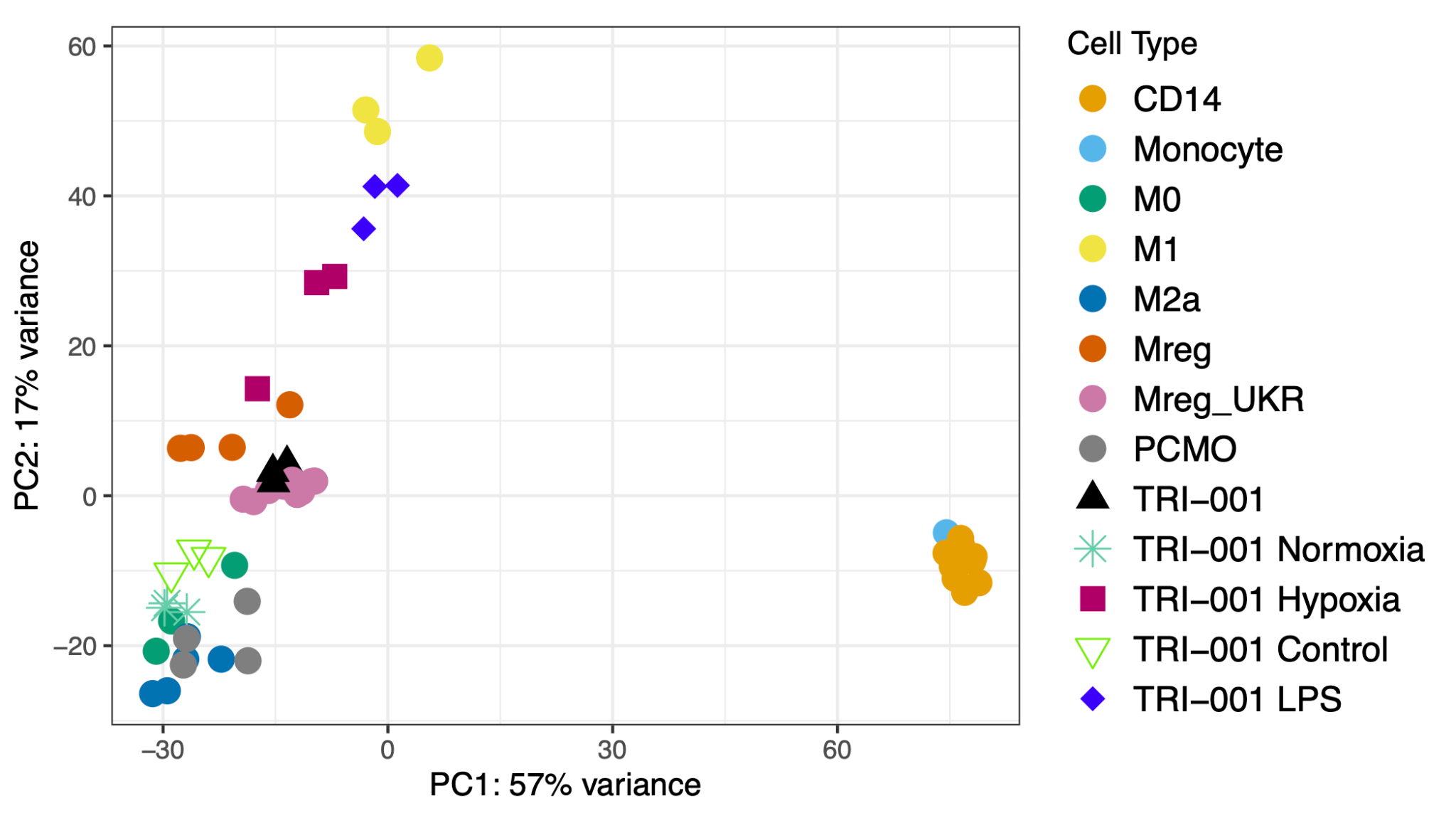*  *Figure S3: Principal Component Analysis (PCA) of the top 500 most variable genes for all samples included in the analysis.*  *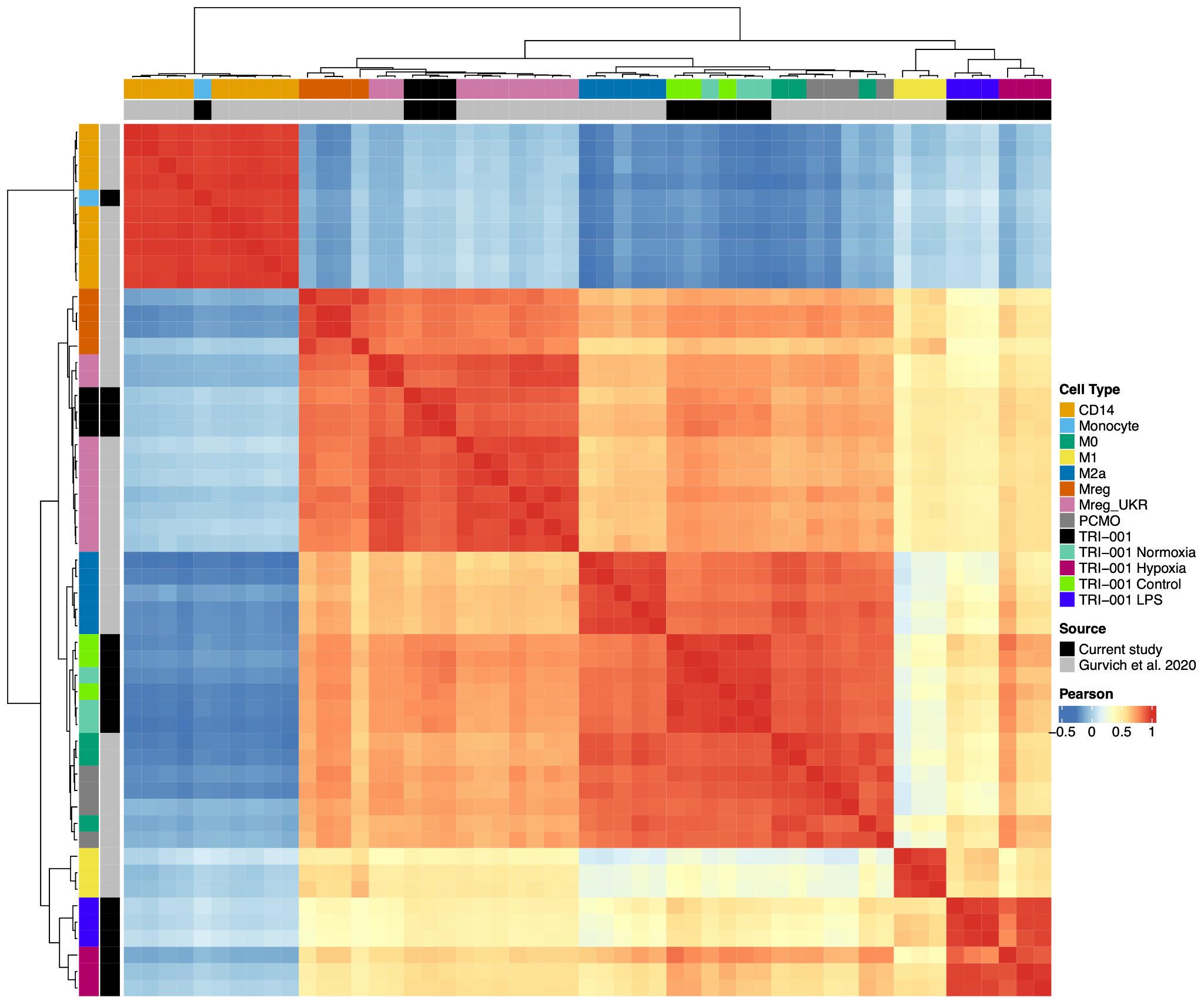*  *Figure S4: Pairwise Pearson correlations for expression of the top 500 genes by variance for all 53 samples included in the analysis.* \| \| --- \| |
| --- | --- | --- | --- | --- | --- | --- | --- | --- | --- | --- | --- | --- | --- | --- | --- | --- | --- | --- | --- | --- | --- | --- | --- | --- | --- | --- | --- | --- | --- | --- | --- | --- | --- | --- | --- | --- | --- | --- | --- | --- | --- | --- | --- | --- | --- | --- | --- | --- | --- | --- | --- | --- | --- | --- | --- | --- | --- | --- | --- | --- | --- | --- | --- | --- | --- | --- | --- | --- | --- | --- | --- | --- | --- | --- | --- | --- | --- | --- | --- | --- | --- | --- |
